# Optical Tissue Phantoms for Quantitative Evaluation of Surgical Imaging Devices

**DOI:** 10.1002/adpr.202200194

**Published:** 2022-10-13

**Authors:** Jason Dinh, Atsushi Yamashita, Homan Kang, Sylvain Gioux, Hak Soo Choi

**Affiliations:** Gordon Center for Medical Imaging, Department of Radiology, Harvard Medical School, Massachusetts General Hospital, Boston, MA 02114, USA; Gordon Center for Medical Imaging, Department of Radiology, Harvard Medical School, Massachusetts General Hospital, Boston, MA 02114, USA; Gordon Center for Medical Imaging, Department of Radiology, Harvard Medical School, Massachusetts General Hospital, Boston, MA 02114, USA; Intuitive Surgical Sàrl, 1170 Aubonne, Switzerland; ICube Laboratory, University of Strasbourg, 67081 Strasbourg, France; Gordon Center for Medical Imaging, Department of Radiology, Harvard Medical School, Massachusetts General Hospital, Boston, MA 02114, USA

**Keywords:** fluorescence-guided navigation, fluorescent phantoms, optical tissue phantoms, tissue-mimicking phantom

## Abstract

Optical tissue phantoms (OTPs) have been extensively applied to the evaluation of imaging systems and surgical training. Due to their human tissue-mimicking characteristics, OTPs can provide accurate optical feedback on the performance of image-guided surgical instruments, simulating the biological sizes and shapes of human organs, and preserving similar haptic responses of original tissues. This review summarizes the essential components of OTPs (i.e., matrix, scattering and absorbing agents, and fluorophores) and the various manufacturing methods currently used to create suitable tissue-mimicking phantoms. As photobleaching is a major challenge in OTP fabrication and its feedback accuracy, phantom photostability and how the photobleaching phenomenon can affect their optical properties are discussed. Consequently, the need for novel photostable OTPs for the quantitative evaluation of surgical imaging devices is emphasized.

## 1. Introduction

Coronavirus disease 2019 (COVID-19) has caused significant shortages in hospital staff, personal protective equipment, and cancellation of nonurgent elective surgical cases. The later outcome of the pandemic has profoundly impacted surgical training globally, limiting trainees’ operative and clinical experiences, and causing the deterioration in their mental health and well-being.^[1]^ Fortunately, optical tissue phantom (OTP) can serve as a temporary solution for this challenge.

**Figure 1.**
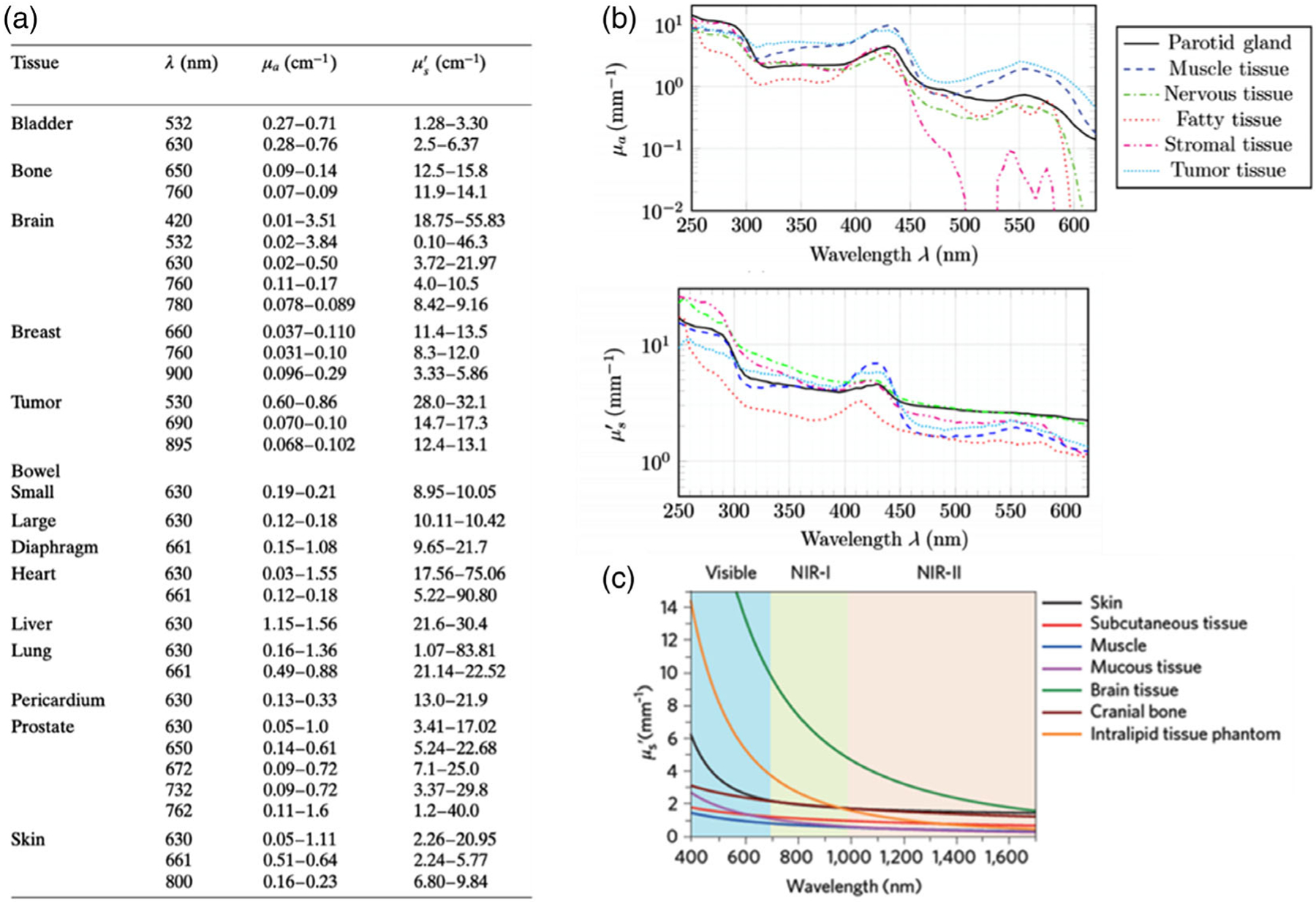
Optical properties of biological tissues and organs. a) In vivo optical properties of various human tissues. Reproduced with permission.^[71]^ Copyright 2011, Wiley-VCH. b) Optical properties of different human glands and tissues. Reproduced under the terms of the CC-BY license.^[72]^ Copyright 2019, The Authors. Published by SPIE. c) Reduced scattering coefficient (*μ*_s_’) of different biological tissues and intralipid scattering tissue phantoms. Reproduced with permission.^[73]^ Copyright 2017, Springer Nature.

OTPs were first introduced in the early 1980s and have quickly expanded their applications to surgical training, medical device evaluation,^[2]^ and ex vivo experiments.^[3]^ The advances in technology in the past decades have translated into a more thorough understanding of biological tissues in terms of their biochemical and optical properties. This deepening knowledge has opened up a potential for utilizing OTPs to emulate the shapes, optical properties, and haptic feedback of various healthy human organs (Figure 1).^[4]^ Several studies reported the construction of phantoms with characteristics similar to those of normal and malignant tissues, such as the breast,^[5]^ prostate,^[2]^ and colon.^[6]^ Moreover, OTPs can be engineered with molecules of specific interests, such as flavin adenine dinucleotide (FAD), nicotinamide adenine dinucleotide (NADH), and collagen, and retain their stability over time and in different conditions.^[7]^ Therefore, these tissue-mimicking phantoms have become realistic and safe media for medical students and surgeons in developing anatomical understanding and practicing surgery.^[2]^ With the increasing demand for surgical precision and safer operations in minimally invasive surgery, OTPs are considered a cost-effective model for quantitative evaluation of surgeon’s performance and image-guided surgical robotic testing.^[8]^ Additionally, tissue phantoms are used to calibrate and validate other clinical instruments, such as dual-energy X-ray absorptiometry (DEXA), magnetic resonance imaging (MRI), computed tomography (CT), and ultrasound imaging.^[9]^

In this review, we explain the essential components of OTPs and introduce the current manufacturing methods and challenges in tissue-mimicking OTPs. We also review the uses of OTPs for surgical applications with a particular focus on photostable near-infrared (NIR) fluorescent phantoms.

## 2. Materials for OTPs

To match tissue properties in OTPs, we must understand the key physical and biochemical characteristics of tissue that influence its interaction with light, such as wavelength dependence, index of refraction, absorption, and scattering.^[7]^ OTPs should be stable over time with easy manufacturing at an inexpensive price. Furthermore, the physical and mechanical properties of OTPs need to be similar with natural tissues for surgical simulation and practice. In general, these crucial parameters of OTPs are separately considered for the selection of bulk materials, absorbing and scattering agents, and fluorophores.

### 2.1. Bulk Matrix

Bulk matrix materials for OTPs can be made in solid and liquid forms. Although liquid OTPs have been used for realistically mimicking tissue spectra, there are several advantages to solid OTPs, such as their long shelf-life, ability to be molded into realistic organ shapes, stable optical properties, and ease of handling in routine instrument validation.^[7]^ Common bulk materials for OTPs include hydrogels, resins, and silicones (Table 1).

Hydrogel-based OTPs represent an intermediate form of solid and liquid. This type of phantom has been produced for various purposes, especially the development of reliable phantoms that accurately mimic the chemical components and mechanical properties of human tissues. Additionally, the compatibility of OTPs in terms of biological and optical feedback is also a critical factor in phantom production. For these reasons, there has been a shift away from solid nonorganic polymers and silicone OTPs, toward biologically compatible materials such as agar, gelatin, and collagen, which can easily contain cellular constituents such as blood or fat, and fluorescent molecules.^[10,11]^ Gelatin and agar are two of the most common examples. These hydrogel-based OTPs allow for the inclusion of organic molecules and cellular-based constituents, but their physical and biochemical stabilities need to be improved significantly. The supplement of formaldehyde to hydrogel-based OTPs can enhance their functions by increasing the matrix’s melting point and maintaining a low Young’s modulus.^[12,13]^ Consequently, this modification makes the phantoms usable at room temperature without the need for refrigeration. Although similar results can also be achieved with agar-based phantoms, this type of OTPs can become fragile under mechanical stress. In terms of biochemical stability, it is possible to fabricate durable OTPs without any bacterial growth for many days and weeks by incorporating toxic species such as wood preservatives,^[14]^ ethylenediaminetetraacetic acid,^[13,14]^ and sodium azide.^[15]^ Similarly, penicillin has also been added to OTPs.^[15]^ Although these additives provide good biological stability, drying out is a concern for the long-term preservation of hydrogel-based OTPs. Therefore, the stability of hydrogel-based OTPs requires further improvement.

Resin and silicone OTPs are solid OTPs, in which physical structures are permanently fixed. These OTPs are manufactured in a similar way by mixing either a resin or silicone with a hardener to create a transparent solid phantom. The entire process can take place within a few days at room temperature or within a few hours at elevated temperatures. Epoxy, polyester, and polyurethane are standard resins for rigid and durable OTPs. In contrast, polydimethylsiloxane (PDMS) is one of the room-temperature-volcanizing (RTV) silicone used for mechanically soft and durable OTPs.^[16,17]^ There are several advantages of RTV silicone for bulk matrix, including its easily tunable texture and optical properties. More specifically, the use of RTV silicone to simulate the mechanical properties of female breast tissue has been investigated by controlling the stiffness of OTPs with different hardener concentrations.^[14,18]^ Compared to rigid resin-based OTPs, soft silicone-based OTPs reduce positioning errors, resulting in smaller standard deviation errors in measuring optical properties.^[14]^ Thus, RTV silicone has been recognized as an excellent bulk matrix of durable OTPs, especially for reproducing mechanical and geometrical properties of human tissues, for example, RTV silicone was used for developing OTPs of rough skin,^[19]^ brain,^[20]^ and tracheobronchial tree.^[16]^

### 2.2. Absorbing and Scattering Agents

**Figure 2.**
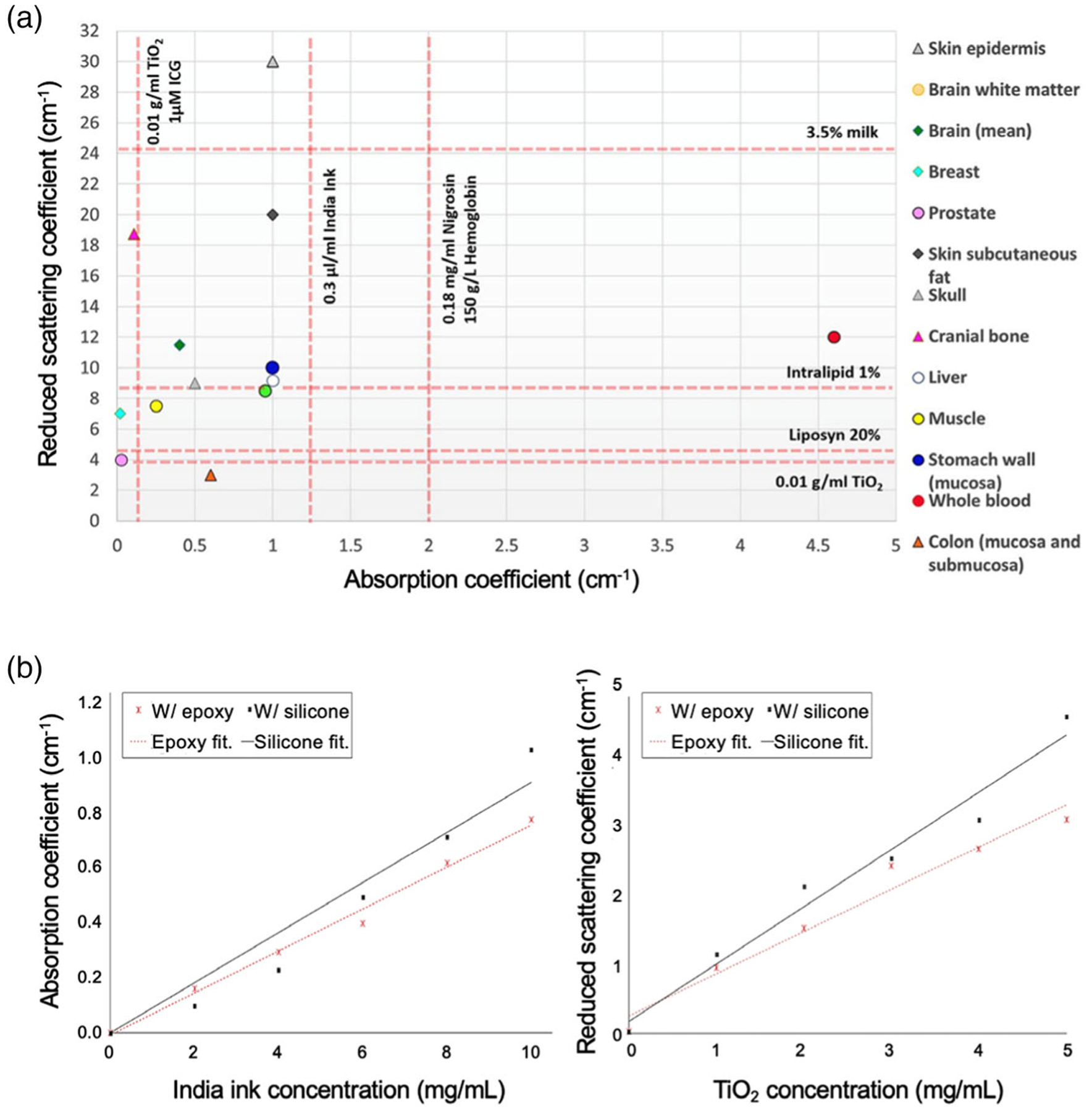
Selection of tissue-specific absorbing and scattering agents. a) Reduced scattering and absorption coefficients of scattering and absorbing agents for tissue phantom fabrication. Reproduced with permission.^[3]^ Copyright 2022, SAGE Publications. b) Absorption coefficient versus India ink concentration and reduced scattering coefficient versus TiO_2_ concentrations. Reproduced with permission.^[74]^ Copyright 2016, SPIE.

Mechanical and geometrical parameters of OTPs are mainly designed by bulk matrix, while the main function of OTPs should be typically designed to reproduce the optical properties (i.e., absorbance and scattering) of biological tissues.^[21]^ Thus, the selection of appropriate absorbing and scattering agents is essential. Vardaki et al. extensively discussed various combinations of agents to simulate the scattering and absorption coefficients of human tissues.^[3]^ In this section, we introduce typical combinations of absorbing and scattering agents to mimic human tissues. Intralipid, liposyn, milk, aluminum oxide (Al_2_O_3_), and titanium dioxide (TiO_2_) are commonly used scatterers while absorbing agents include different types of inks, biological absorbers, and fluorophores. Metal oxide powders are cost-effective scattering agents, especially TiO_2_ has been most commonly used for several types of bulk materials such as resin, silicone, and oil.^[22]^ Figure 2a shows typical concentrations of scattering and absorbing materials compared to the optical properties of various biological tissues.^[3]^ Mixing scattering and absorbing agents in specific proportions can generate phantoms with desirable optical properties. For example, prostate-mimicking OTPs can be fabricated with 0.01 g mL^−1^ of TiO_2_ and approximately 1 μm of indocyanine green (ICG) as scattering and absorbing agents, respectively.

Additionally, Liu et al. quantitatively examined the correlations between particle concentrations and optical properties of phantoms.^[21]^ As shown in Figure 2b, direct proportionality was observed between the concentrations of India ink and TiO_2_, and absorption (*μ*_a_) and reduced scattering (*μ*_s_’) coefficients, respectively. These optical properties were calculated at 650 nm and fitted using a linear fit generated with origin curve fitting. Based on the findings, optimal amounts of TiO_2_ and India ink can be determined to reproduce the properties of the epidermis and dermis layers of the skin.^[21]^

### 2.3. Fluorophores

**Figure 3.**
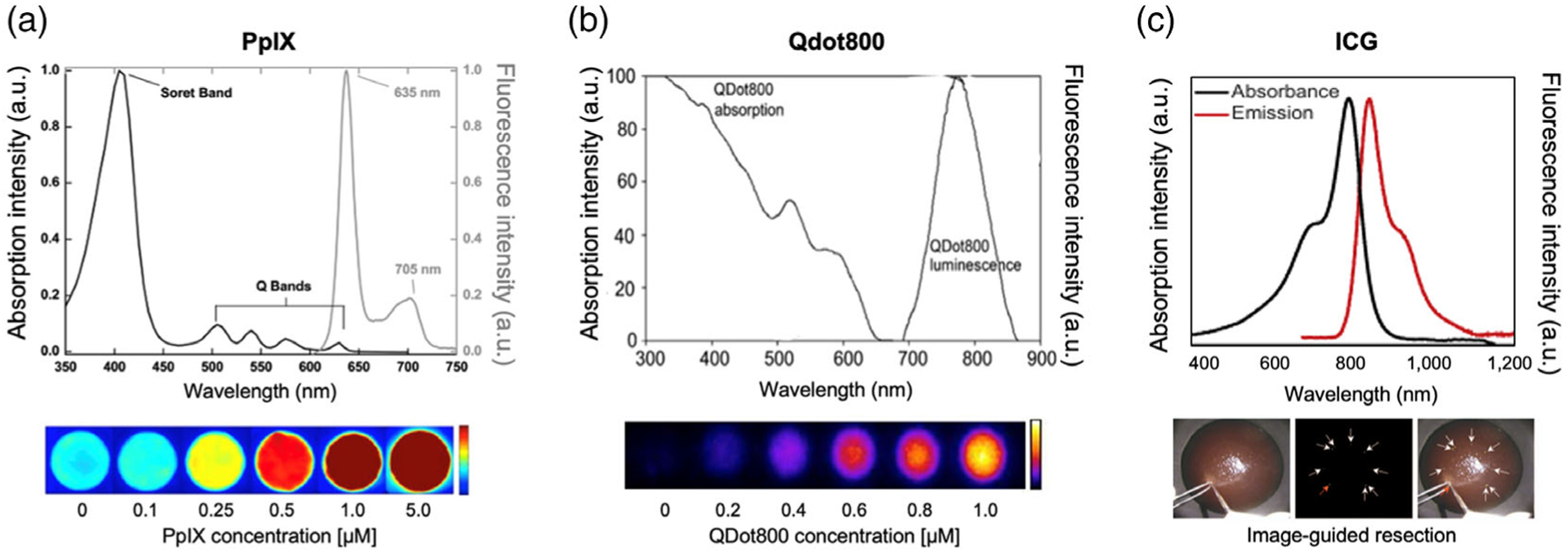
Optical properties of fluorophores used for OTP fabrication. a) PpIX and background-subtracted images from the pulse-imaging system in ambient light with different concentrations of PpIX. Reproduced with permission.^[75]^ Copyright 2013, Optical Society of America. b) Qdot800 (reproduced with permission.^[76]^ Copyright 2012, IOP Science) and fluorescence images acquired from different Qdot800-based phantoms (reproduced with permission.^[25]^ Copyright 2014, Wiley-VCH). c) ICG^[77]^ and NIR fluorescence image-guided resection of ICG beads.^[26]^ Reproduced with permission.^[26]^ Copyright 2006, SPIE.

The addition of fluorescent components to phantoms can improve their optical feedback at different wavelengths and light conditions. Consequently, fluorescent OTPs can be used to track the performance of fluorescence imaging instruments, including robotic surgical systems.^[2]^ Some frequently used fluorophores are protoporphyrin IX (PpIX), Quantum dot 800 (Qdot800), and ICG. With their optical properties in the visible spectrum, PpIX fluorescent phantoms have been utilized to demonstrate fluorescence-guided spectroscopic margin delineation^[23]^ and evaluate novel fluorescence-guided surgery systems under normal room light.^[21]^ As illustrated in Figure 3a, PpIX allowed surgeons to visibly detect fluorescence at low concentrations of fluorophore under room light conditions. Despite this advantage, PpIX can easily suffer from photobleaching due to its sensitivity to intense background illumination of the operating room.^[24]^ Alternatively, endogenous photobleaching resistant alternatives, such as FAD and NADH, have been used as visible fluorophores to characterize a pulsed, multiwavelength system under strong ambient illumination conditions.^[24]^

In contrast, due to the tissue-penetrating characteristics of NIR fluorophores, Qdot800 and ICG phantoms have been used to test the NIR fluorescence imaging system^[25]^ and surgeon’s training in image-guided surgery (Figure 3b,c).^[26]^ More specifically, despite its lack of tissue-specificity, Qdot800 has been incorporated into OTPs to evaluate the performance of charged-coupled device (CCD) cameras,^[25]^ quantify excitation light leakage, and assess the sensitivity of the NIR fluorescence imaging device.^[27]^ Due to its photostability, Qdot800-integrated phantom enables optimizing imaging devices and clinical translation of NIR imaging agents.^[25]^ In contrast, tissue-specific studies have used ICG phantoms, including training surgeons in breast-conserving surgery^[8]^ and assessing fluorescence-detecting systems in sentinel lymph node mapping.^[28]^

## 3. Fabrication of OTPs

The structural design of solid OTPs is strongly influenced by their intended purpose. For training and simulating therapeutic purposes, such as image-guided surgery, photodynamic therapy (PDT), and photothermal therapy (PTT), the physical and mechanical properties of phantom should be fabricated to best imitate patient tissues.^[14,16,18]^ For testing and calibrating optical machines, an OTP with a simple shape and convenient size for handling would be prioritized design features. However, other design features still need thorough consideration in the manufacturing of OTPs.^[29]^ For example, the phantoms need to be optically reproducible and homogenous while maintaining a linear optical behavior over a broad concentration range of absorbing and scattering agents. In this section, we describe manufacturing methods for structure-mimicking OTPs.

### 3.1. Molding

**Figure 4.**
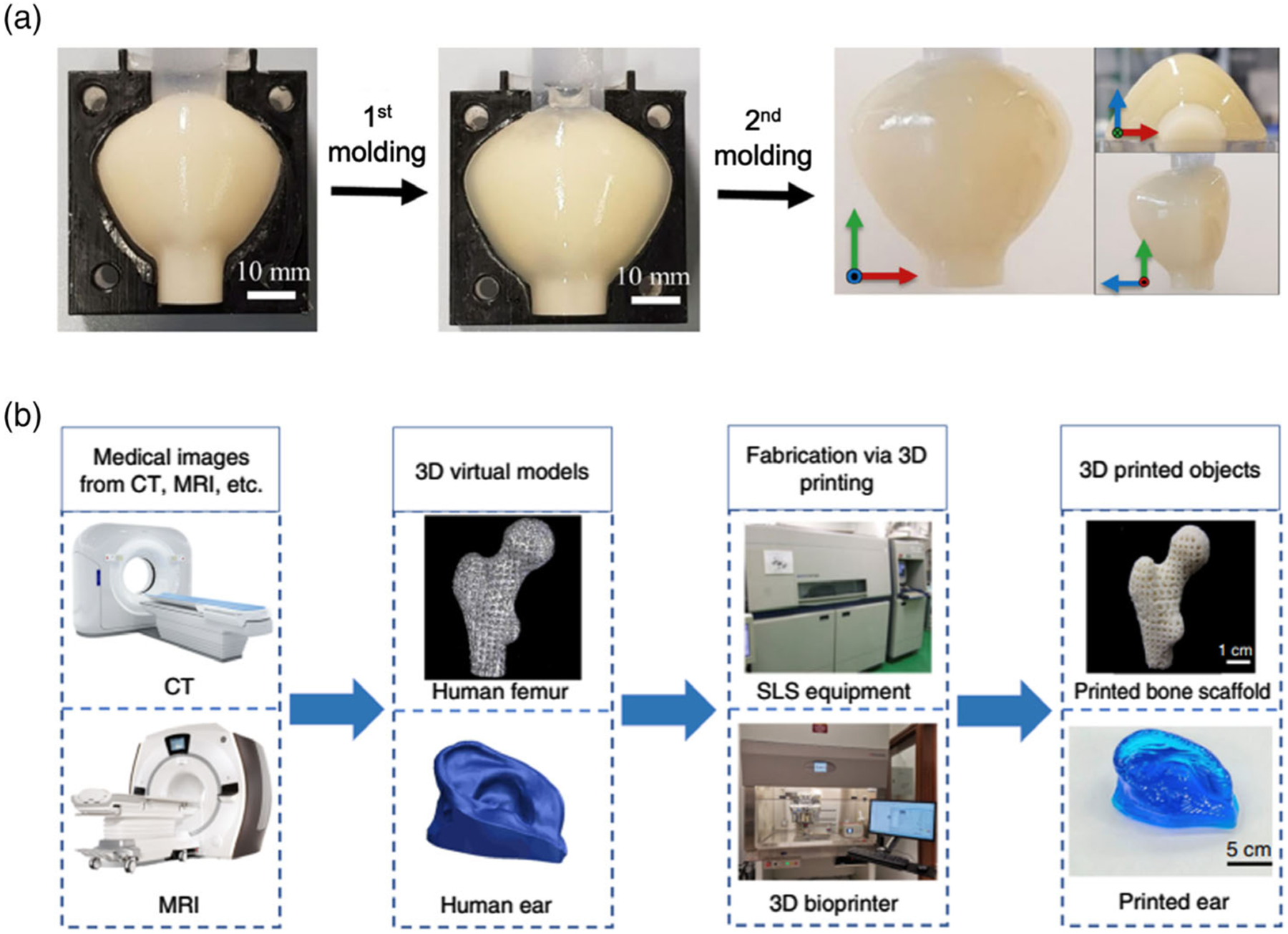
Manufacturing methods of OTPs. a) Molding process of prostate phantoms. Reproduced with permission.^[2]^ Copyright 2019, Springer Nature. b) A typical fabrication process for 3D printing of biomedical products. Reproduced with permission.^[33]^ Copyright 2021, AIP Publishing.

Molding is a conventional manufacturing method for OTP fabrication, which can be used on several types of bulk matrixes, including hydrogel, resin, and RTV silicones (Figure 4a).^[30]^ For mass production of uniformly shaped OTPs, molding is the most economical way. In this method, bubbles inside a phantom are eliminated by degassing. Additionally, heating^[6,26]^ and chemical reactions^[12,13]^ are required to successfully solidify the phantom. Metals have also been widely used for phantom fabrication.^[20]^ However, such molds are limited to simply shaped phantoms due to the difficulties in manipulating the shape of metals. To create more reliable phantoms for tissue imaging and therapeutic plans, there has been an increasing demand for the development of more advanced and malleable molds. For instance, tracheobronchial tree OTP was developed by casting a negative tissue imprint in wax and then immersed it in silicone.^[16]^ After the curing process, the wax was removed by melting. However, this method can produce only one mold at a time and requires the resection of the actual organs. Thus, this is not suitable for creating OTPs for many patients.

### 3.2. Three-Dimensional (3D) Printing

Recently, a more advanced method is the development of phantom molds via 3D printing using patients’ imaging data (Figure 4b). This technique can produce both the phantom and the mold for different types of OTPs.^[6,31]^ In this approach, acrylic-based photopolymer, polyvinyl acetate, and cyclic olefin polymer were adopted for the matrix. For the fabrication of structurally complicated OTPs, 3D printing is preferred over the traditional molding method.

3D printing has been broadly used in the applications of biomanufacturing, enabling the printing of realistic anatomical human organs from medical images such as CT and MRI.^[32,33]^ This method has several advantages, including a short production cycle and the ability to make phantoms with complex geometric characteristics and internal structures.^[34]^ As tissue features and pathologies are different across patients, the 3D printing method is suitable for fabricating tissue-simulating OTPs tailored to specific patients. Recent studies have reported the uses of 3D-printed phantoms for CT, MRI, mammography, single-photon emission CT (SPECT), positron emission tomography (PET), ultrasound, Raman spectroscopy, and radiation therapy.^[3,35–37]^ Larsson et al. developed a 3D phantom of an infant’s upper body with the lungs, heart, bones, and muscles.^[38]^

Although various types of 3D-printed tissue phantoms have been reported in recent years, only a limited number of studies discussed NIR OTPs. One of the reasons may be the limitation of compatible NIR fluorophores. Nevertheless, this drawback can be overcome by mixing the dyes with epoxy,^[39]^ acrylonitrile butadiene styrene,^[40]^ resins, or liquids of scattering agents.^[39]^ Such methods were applied for creating 3D-printed brains^[41]^ as well as upper body^[38]^ NIR phantoms. Additionally, PDMS is an optically clear polymer that can be readily combined with scattering and absorbing particles with a higher level of tunability.^[7,42]^ Importantly, 3D printing allows for the formation of high-quality calibrated OTPs.^[43,44]^ By manufacturing OTPs with known properties and surface geometry, component-by-component calibration of the measured data becomes possible. However, it is still challenging to fabricate OTPs that can accurately mimic both the optical and structural properties of biological tissues.^[32,45–47]^ In some cases, high temperature^[40,48]^ can cause unwanted degradation of fluorophores. Meanwhile, photopolymer-based printing systems, including stereolithography^[41,49]^ and material jetting techniques,^[50–52]^ have limited the tuning of optical properties and printing parameters.

## 4. Photostability of OTPs

Due to their vast applications in imaging system testing and fluorescence-guided surgery, OTPs must retain their optical properties throughout extended irradiation. Consequently, there is an urgent need for photostable OTPs with optimal concentrations of scatterers, absorbers, and fluorophores. In the following sections, we will discuss the photobleaching phenomenon of fluorophores and evaluate a promising photostable alternative to ICG.

### 4.1. Photobleaching

**Figure 5.**
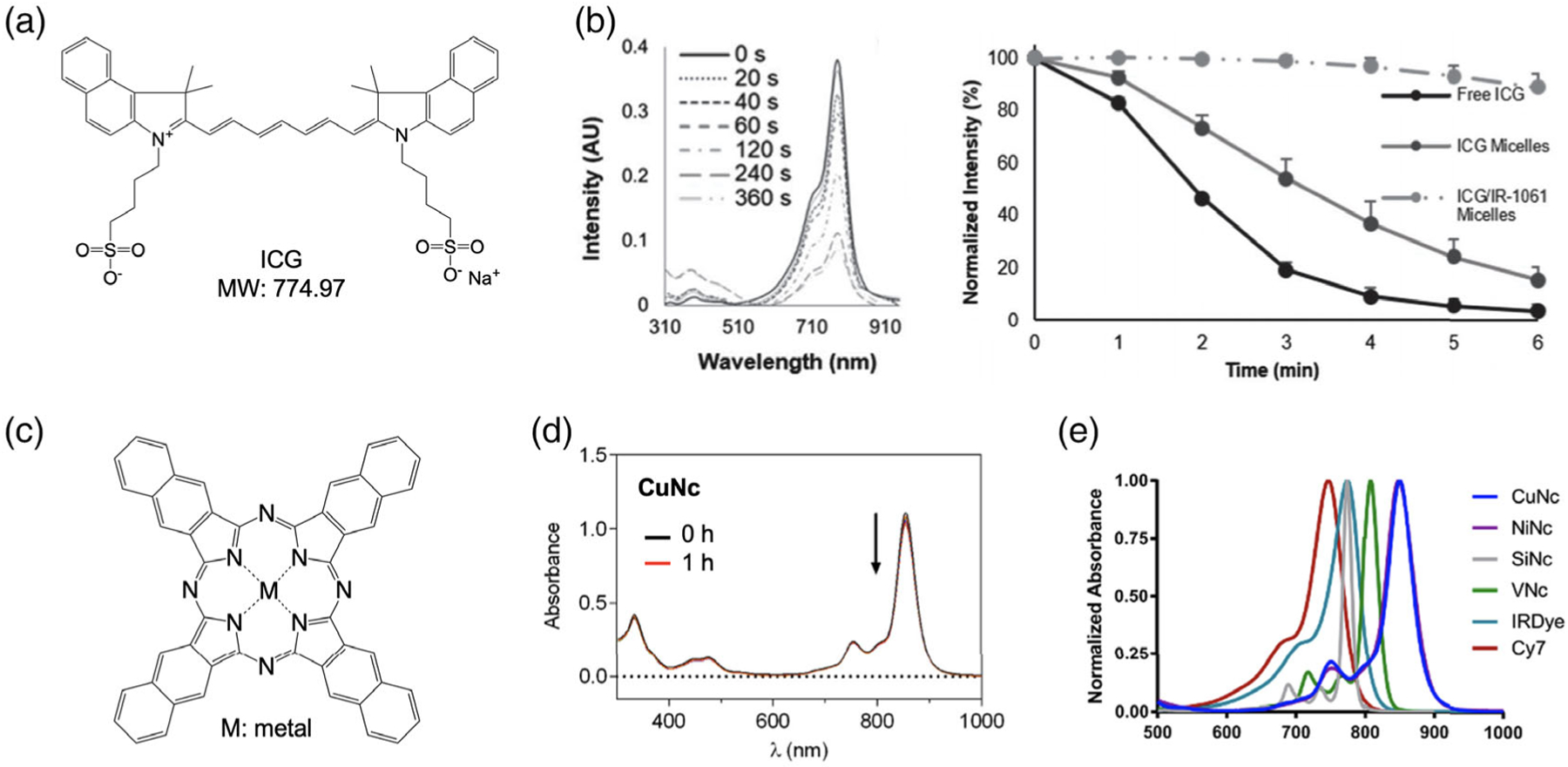
NIR fluorophores for OTPs. a) Chemical structure of ICG and b) optical stability in water (3 μm) compared with micelles. Reproduced with permission.^[58]^ Copyright 2019, Technical Association of Photopolymers. c) Chemical structure of naphthalocyanines. d) Optical stability of CuNc upon NIR irradiation (*λ* > 715 nm) of a sample up to 1 h, and e) absorption spectra (5 μm) of various naphthalocyanines compared with IRDye and Cy7. Reproduced with permission.^[59]^ Copyright 2018, Elsevier.

Photobleaching is the irreversible extinction of fluorophores upon continuous exposure to photoexcitation, resulting from an enhanced reactivity of the fluorophores in their excited states.^[53]^ ICG is a U.S. Food and Drug Administration (FDA)-approved NIR fluorophore and has been commonly used in image-guided cancer surgery due to its tumoral uptake in microdose concentrations (Figure 5a).^[54]^ However, ICG suffers from photobleaching upon extended exposure to exciting light sources.^[55–57]^ Yeroslavsky et al. examined the decomposition of ICG into smaller nonfluorescent molecules after being irradiated with an 808 nm laser at 2.8 W.^[58]^ This degradation is manifested as a decline in ICG’s absorption peak at 780 nm and fluorescent intensity after 6 min (Figure 5b). Due to the photoinstability of ICG, additional amounts of fluorescent dye may be needed during extended surgical procedures to allow surgeons to accurately locate targeted tissues.

With their robust multiring structure containing a central metal (Figure 5c), naphthalocyanine dyes can overcome the set-back of photobleaching in ICG. When exposing naphthalocyanine fluorophores to NIR irradiation up to 1 h, Duffy et al. illustrated the ability of these molecules to effectively retain their absorbances (Figure 5d).^[59]^ Additionally, as their absorption spectra are in the range of 760–850 nm depending on their central metal and types of substituents (Figure 5e),^[59]^ these types of fluorophores offer potential ultrastable alternatives to ICG in real-time NIR fluorescence-guided surgery. Furthermore, the photobleaching resistance of naphthalocyanine is demonstrated in the concurrent NIR fluorescence imaging and combinatorial phototherapy.^[60]^ Interestingly, the central metals of naphthalocyanine fluorophores enable the detection of visible emissions through the energy transfer of core ions. This dual visible-NIR emission property of naphthalocyanine facilitates accurate laser beam placement during NIR fluorescence-guided tumor resection.^[61]^

### 4.2. Photostability Test

**Figure 6.**
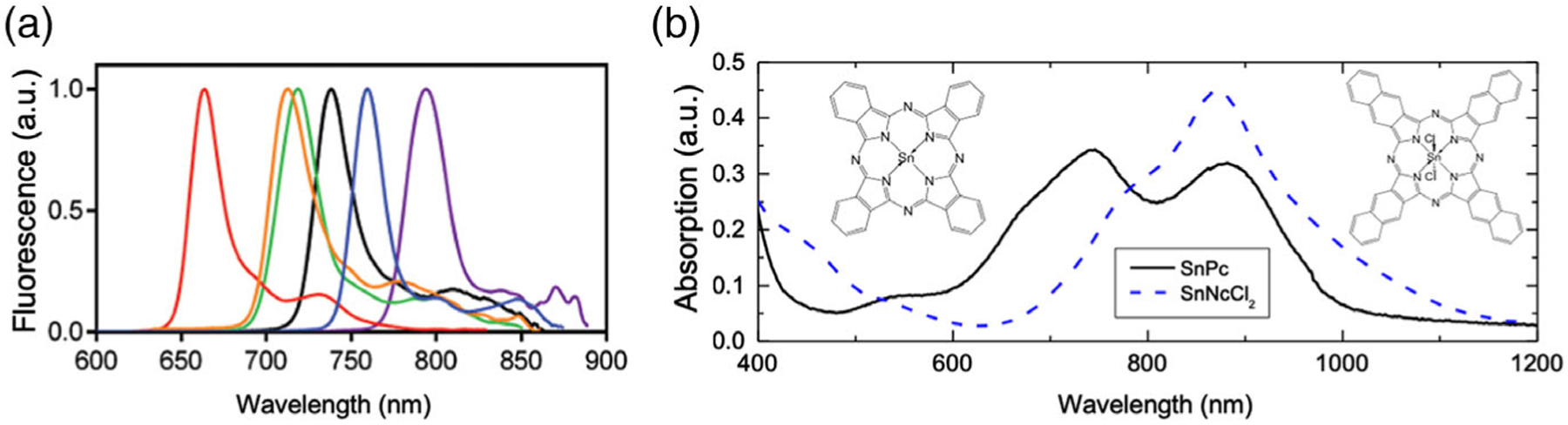
Optical properties of phthalocyanines and naphthalocyanines. a) Normalized fluorescence spectra of phthalocyanine (green), naphthalocyanine (magenta), and their aza-analogs. Reproduced with permission.^[78]^ Copyright 2015, Royal Society of Chemistry. b) Thin film absorbance of tin naphthalocyanine dichloride and tin phthalocyanine. Reproduced with permission.^[79]^ Copyright 2013, Elsevier.

The photostability of tin (IV) 2,3-naphthalocyanine dichloride (SnNc–Cl_2_) was evaluated against ICG due to its similarity in absorbance and fluorescence. In fact, when comparing naphthalocyanine to its phthalocyanine counterpart, the optical properties of SnNc–Cl_2_ closely match those of ICG (Figure 6).

**Figure 7.**
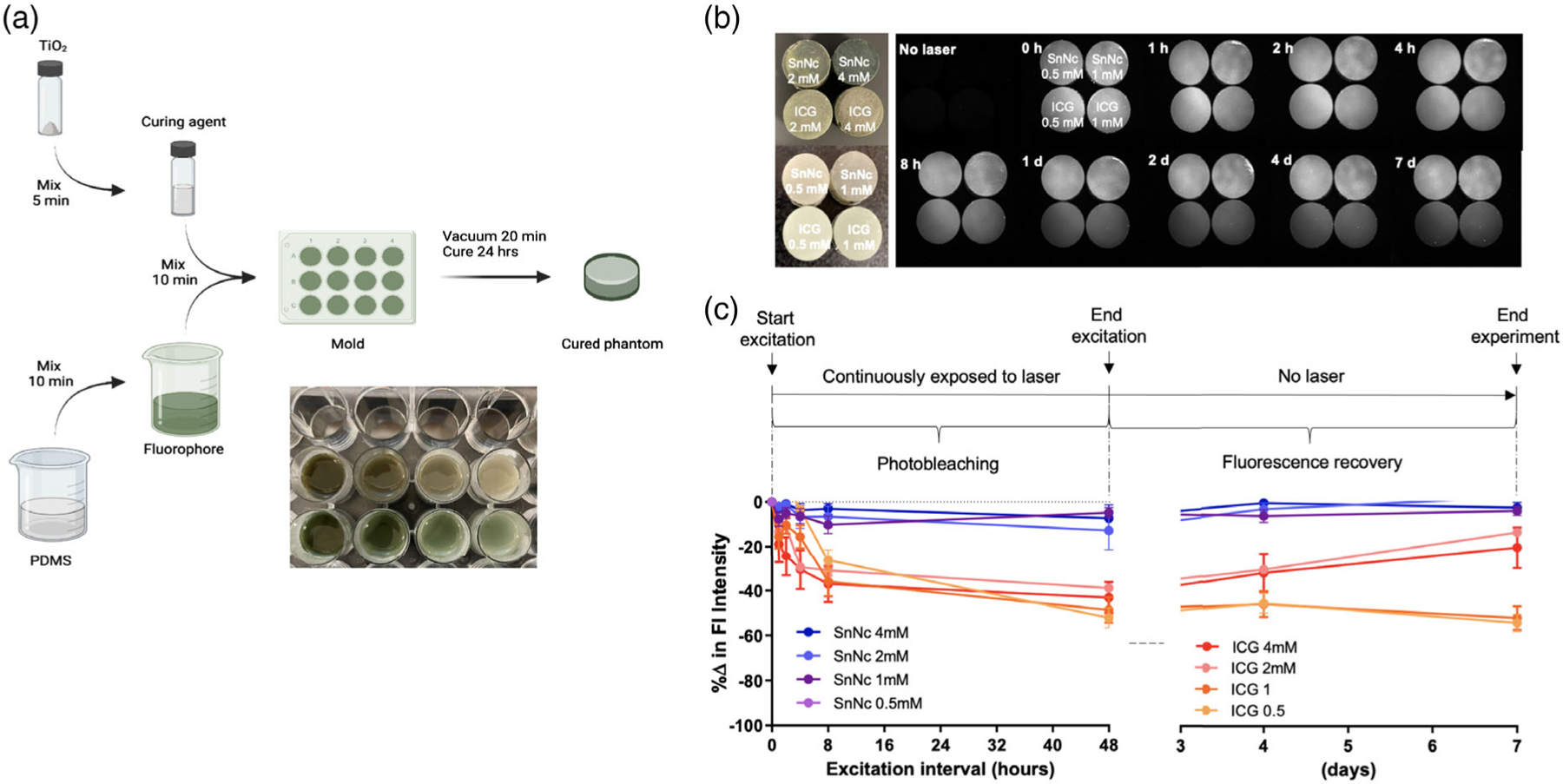
Fabrication and optical properties of photostable OTPs. a) Fabrication of silicone phantoms using SnNc–Cl_2_ and ICG. b) NIR fluorescence (Fl) images, and c) Fl intensity of phantoms after continuous laser exposures.

For photostability tests, silicone phantoms of SnNc–Cl_2_ in toluene, chloroform, dichloromethane, or combinations of two solvents were compared with ICG samples with corresponding concentrations and solvents. Initially, 2.22 mm SnNc–Cl_2_ and ICG in transparent glass containers were continuously exposed under 808 nm excitation at 35 mW. After the optimal solvent (toluene) was determined, samples with different concentrations (0.5, 1, 2, and 4 mm) of SnNc-Cl_2_ and ICG were similarly exposed under the excitation laser continuously for 2 days, and the fluorescence recovery was reinvestigated on day 4 and day 7 postexcitation (Figure 7a).

As shown in Figure 7b, SnNc–Cl_2_ phantoms at any concentrations (0.5, 1, 2, and 4 mm) showed better photostability than ICG at the corresponding concentrations during the entire study period (7 days). Interestingly, among the tested, 0.5 mm SnNc–Cl_2_ showed the best photostability with an improved fluorescence intensity compared to its original intensity after being exposed to the 808 nm excitation, suggesting that quenching may have occurred at higher concentrations of SnNc–Cl_2_. Although some fluorescence of ICG phantoms at 2 and 4 mm remained after 48 h of continuous laser exposure, the recovered fluorescence intensities were still lower than those of all SnNc–Cl_2_ samples on day 7, indicating that SnNc–Cl_2_ is more photostable than ICG (Figure 7c). Although previous studies showed that photobleaching was permanent in ICG and its fluorescence intensity could not be recovered after extended excitation exposure,^[62]^ our finding demonstrated that ICG’s fluorescence could be restored when high concentrations (>2 mm) of ICG were incorporated into silicone phantoms. Due to their unique electronic structures, metal naphthalocyanine complexes absorb and emit light in the NIR window and are characterized by superior photostability and structural integrity under extensive photoirradiation and NIR laser exposure. Indeed, SnNc–Cl_2_ silicone phantoms offered highly stable fluorescence that was minimally affected by the 808 nm excitation over the 7 day testing period, while the commonly used ICG showed greatly decreased fluorescence due to photobleaching.

## 5. Surgical Applications of Fluorescent OTPs

**Figure 8.**
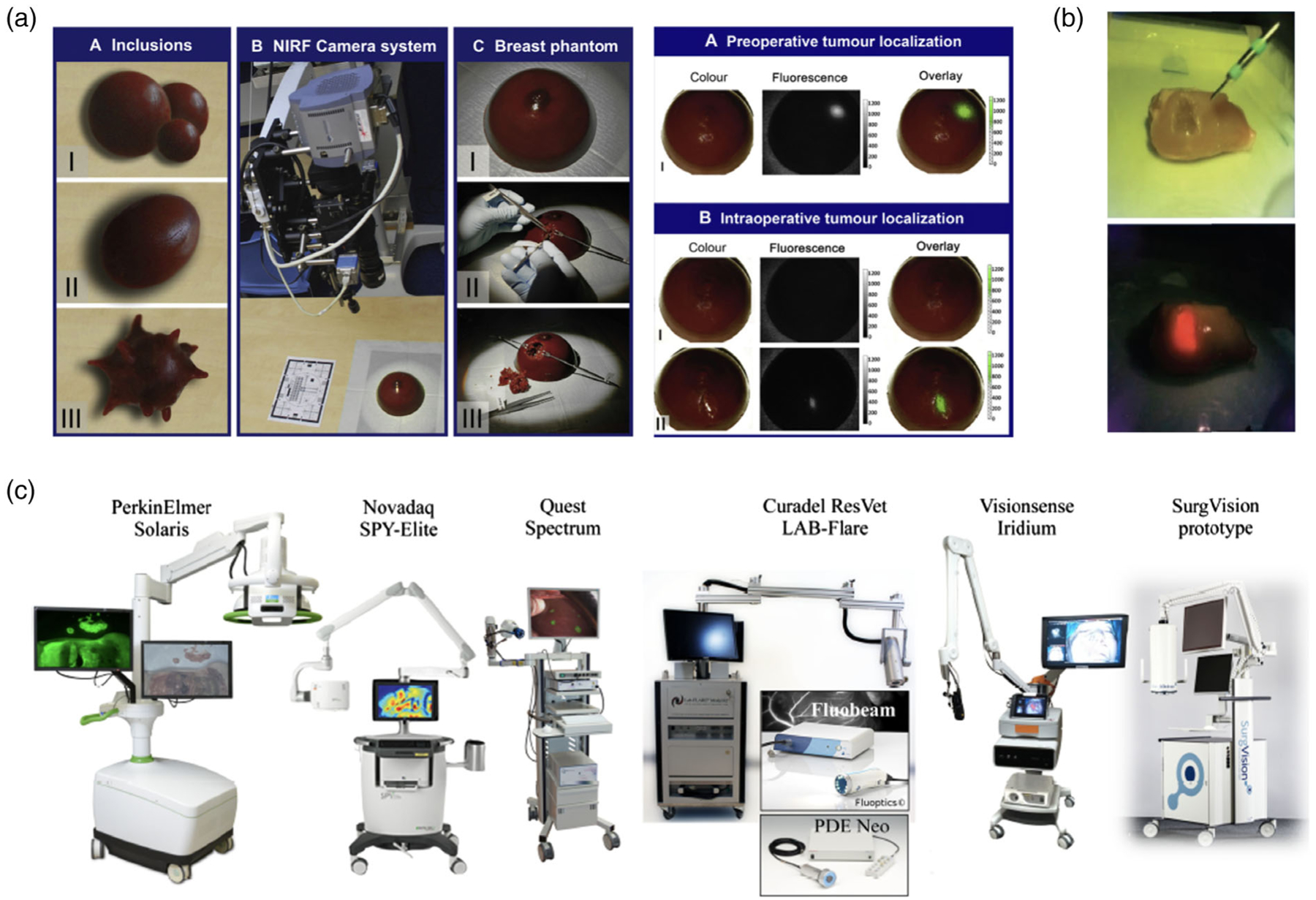
Surgical applications of OTPs. a) Integration of fluorescent tumor-like agarose inclusions in breast-shaped phantoms and real-time NIR-guided tumor resection. Reproduced with permission.^[8]^ Copyright 2011, Elsevier. b) Imaged-guided spectroscopic margin delineation of an OTP inserted into ex vivo chicken muscle tissue. Reproduced under the terms of the CC-BY license.^[23]^ Copyright 2021, SPIE. c) Fluorescence imaging systems for surgery. Reproduced under the terms of the CC-BY license.^[80]^ Copyright 2016, SPIE.

With the development of tumor-targeting contrast agents,^[63–65]^ fluorescent OTPs can be manufactured for ex vivo experiments. ICG has widely been used for fabricating tumor-simulating phantoms as inclusions in agarose breast phantoms (Figure 8a).^[8]^ By using fluorescent tumor-like inclusions differing in size and shape, Dam et al. were able to assess tumor localization, both preoperatively and intraoperatively, and evaluate the effectiveness and sensitivity of NIR fluorescence-guided tumor resection.^[8]^ Furthermore, Horgan et al. utilized a PpIX phantom inserted into ex vivo chicken muscle tissue to investigate the efficacy and accuracy of margin delineation of fluorescence-guided spectroscopy (Figure 8b).^[23]^

Beyond training surgeons to use intraoperative fluorescence technology, fluorescence phantoms will play a key role in standardizing the technology across imaging systems and health care professionals.^[66–69]^ Indeed, because existing fluorescence imaging systems differ in design, their performances vary, and the results obtained cannot be compared between surgical cases, systems, and users. To overcome this limitation, fluorescence phantoms can be used to assess system performances and calibrate imaging systems to obtain data that is more comparable.^[52]^ In particular, one of the key challenges in the near future will be the understanding and interpretation of fluorescence values to guide decision-making (“where do I cut?”) as well as deriving a consensus internationally among surgeons, hospitals, and imaging systems.^[70]^ Such consensus will only be possible if imaging systems provide comparable results enabling the determination of threshold values in specific surgical procedures. Fluorescence phantoms have a strong potential to help achieve this longstanding objective, along with the proper training of surgeons, the development of standardized surgical procedures, and the development of fluorescence imaging methods that are more quantitative in nature.

## 6. Conclusion and Outlook

Despite its widespread applications, ICG phantoms suffer from undesirable photoinstability upon irradiation, which can affect their ability to provide accurate fluorescence feedback. This limitation of OTPs calls for more in-depth studies on NIR photostable candidates for phantom fabrication. These potential alternatives should be able to sustain their tissue-like optical properties while advancing the advantages of NIR fluorophores, including reduced signal scattering and deeper tissue penetration. Moreover, as more knowledge of the optical properties of human tissues is obtained, there is a constant need for novel materials for OTPs that can closely reproduce conditions inside the human body.

As the field of fluorescence-guided surgery is moving closer to clinical validation and adoption, with newer specific contrast agents becoming available, the need for standardization across imaging systems will become more pressing. As for any medical image-based guidance method, a professionally driven consensus will need to be established along with the definition of standard surgical procedures. Fluorescence phantoms will play a significant role in establishing such a consensus by helping calibrate imaging systems to ensure that proper, robust, and comparable performance is obtained between manufacturers. Fluorescence phantoms will also help in providing training to surgeons in using this emerging technology.

## Figures and Tables

**Table 1.** Properties of materials for OTP matrix and their recommended use.^[7,42]^

Matrix material	Form	Physical stability	Refraction index	Recommended use
Aqueous suspension	Liquid	–	1.34	Multiple phantom contrast studies
Gelatin hydrogel	Flexible	Fragile	1.35	Detailed heterogeneity phantom studies bioabsorbers and fluorophores
Agar hydrogel	Flexible	Fragile	1.35	Detailed heterogeneity phantom studies bioabsorbers and fluorophores
Epoxy resin	Solid	Permanent	1.54	Calibration and routine validation Intersystem comparison
Silicone	Flexible	Permanent	1.4	Complex geometries with permanent flexible phantoms
